# How Strong Is the Link Between Merkel Cell Carcinoma and the Occurrence of Other Skin Cancer Types? A Meta‐Analysis

**DOI:** 10.1111/exd.70092

**Published:** 2025-03-26

**Authors:** Trairong Chokwassanasakulkit, Nigel A. J. McMillan

**Affiliations:** ^1^ Institute of Biomedicine and Glycomics and School and Pharmacy and Medical Sciences Griffith University Gold Coast Australia

**Keywords:** geographic areas, melanoma, Merkel cell carcinoma, non‐melanoma skin cancer, second skin cancer

## Abstract

This meta‐analysis presents findings from nine studies involving 4626 cases of primary Merkel cell carcinoma (MCC), aimed at evaluating the relationship between primary MCC and the incidence of a second skin cancer. The analysis reveals a significant association, with a calculated risk ratio of 2.97 (95% CI, 1.70–5.19, *p* = 0.0001), indicating that individuals diagnosed with primary MCC are nearly three times more likely to develop the second skin cancer compared to patients with other second cancers. Among the second skin cancers analysed, basal cell carcinoma (BCC) showed the highest risk (0.69, 95% CI 0.35–1.37), followed by squamous cell carcinoma (SCC) (0.45, 95% CI 0.23–0.90) and melanoma (0.31, 95% CI 0.19–0.50). While geographic analysis showed that patients in Northern Europe have a non‐significant 1.7‐fold increased likelihood of developing the second skin cancer relative to those in North America (USA), where the likelihood of developing the second skin cancer is significantly lower at 0.7 times. The results underscore the importance of implementing enhanced surveillance and preventive strategies for individuals at increased risk. By identifying these associations, we may improve the early detection of the second skin cancer in patients with MCC.

## 1 Introduction

Merkel cell carcinoma (MCC) is a rare, aggressive cutaneous malignancy, characterised by rapid growth and aggressive nature. MCC results in poor survival rates and a high recurrence rate of approximately 40% [1]. Particularly concerning is its low 5‐year overall survival rate in advanced stages, and MCC patients diagnosed at stage IV have a median survival duration of merely 14 months [2]. MCC predominantly affects older adults, especially those with lighter skin tones and individuals with weakened immune systems. In recent decades, there has been a significant increase in the incidence of MCC, and this trend is expected to continue [3]. However, translating preclinical research into effective clinical strategies presents challenges. For instance, there is a lack of diverse MCC cell lines, and existing lines can be difficult to work with in vitro, such as challenges in transfecting the MCC cells [4]. Additionally, finding a transgenic mouse model to study the role of MCPyV T antigen in MCC progression poses significant challenges. The diagnostic process can also be complicated by some false‐negative results, which impede the accurate evaluation of patients with MCC [5, 6].

As a result of these challenges, there is a critical need for enhanced studies into the aetiology, risk factors and associations with other skin cancers. Such investigations are essential to developing effective treatment strategies and potential cures for patients diagnosed with MCC. Emerging evidence suggests that MCC may not be an isolated occurrence, but rather develop in conjunction with other skin malignancies [7]. MCC patients could potentially develop a genetically distinct “second primary” MCC, likely through similar pathogenic mechanisms. Studies have also revealed that MCC is associated with a higher incidence of “second skin cancer” beyond second primary MCC, including melanoma and non‐melanoma skin cancers (NMSC) such as squamous cell carcinoma (SCC) and basal cell carcinoma (BCC) [8]. These “second cancers” are distinguished from “secondary cancers” or “metastases from primary cancers”, which represent local MCC recurrences, based on genetic variations identified through genomic studies, such as chromosomal copy number changes [9]. Secondary cancers are new malignancies that develop as a result of the original cancer or its treatment, whereas second cancers refer to a new primary malignancy that develops over a 1‐ to 5‐year period following diagnosis compared to the general population [10].

Despite escalating interest in this connection, the strength of the association between primary MCC and a second skin cancer remains ambiguous. Variability in findings may be influenced by factors such as genetic predisposition, compromised immune responses, shared precursors in the skin and the concurrent appearance of different malignant neuroendocrine tumours [11]. Disparities in research methodologies, patient demographics and geographical contexts also contribute to these inconsistencies, further emphasising the necessity of a comprehensive review of existing literature to clarify the potential risks of second skin cancer in MCC patients.

This meta‐analysis aims to systematically synthesise data from multiple studies to quantify the risk of developing second skin cancer in individuals diagnosed with primary MCC. By bringing together findings from various geographic regions and demographic backgrounds, we seek to identify the geographic trends and risk factors associated with different types of second skin cancer. Through this analysis, we seek to highlight the necessity of vigilant monitoring and preventive strategies for patients with primary MCC, especially given the observed geographic variations in skin cancer incidence and the impact of each type of second skin cancer. Understanding these associations can lead to the development of improved screening protocols, enhanced early detection methods and tailored treatment strategies, ultimately resulting in better patient outcomes for those affected by primary MCC.

## 2 Methods

### 2.1 Data Sources and Searches

**FIGURE 1:**
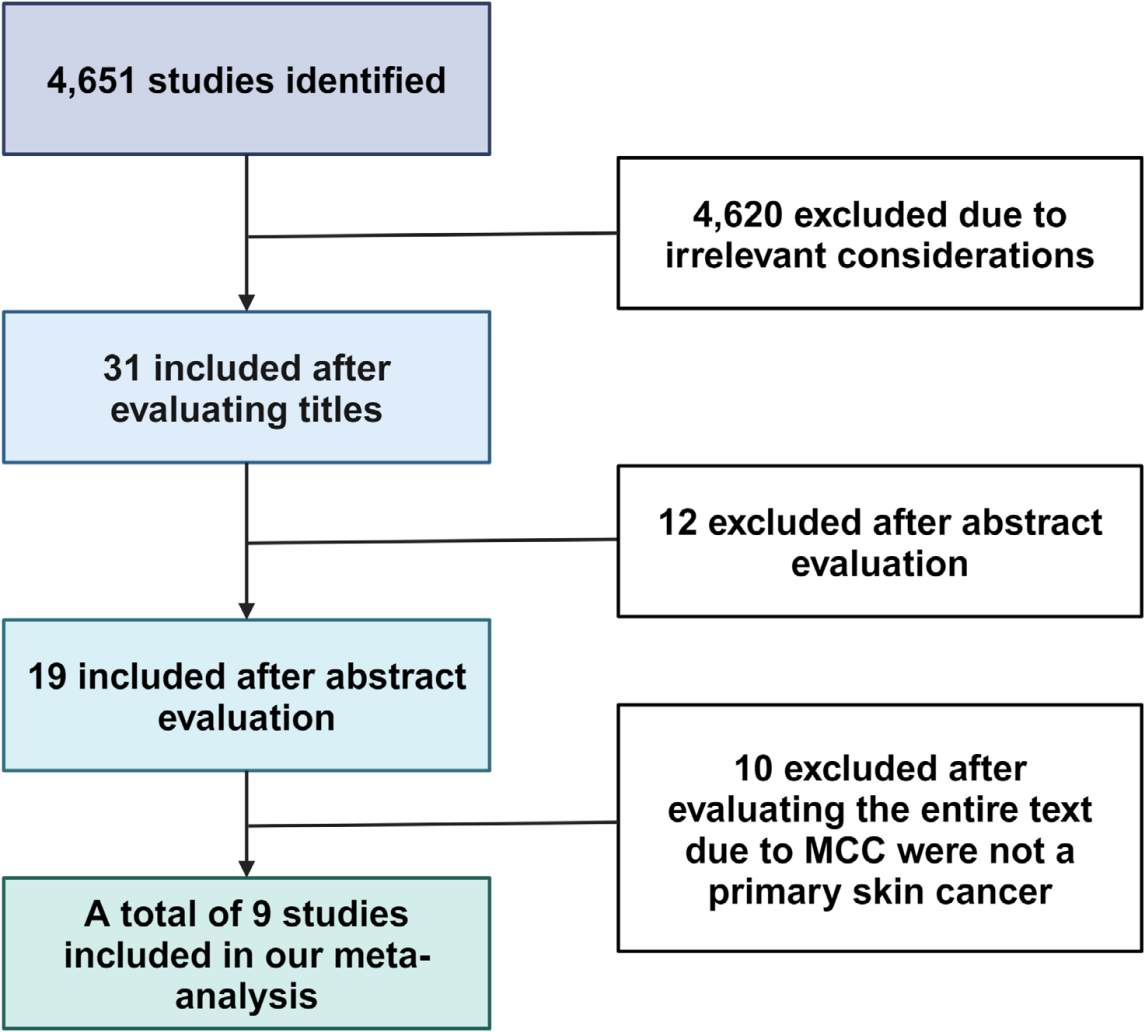
A PRISMA schematic representation of the process used to select studies for inclusion in a meta‐analysis, consisting of multiple sequential stages.

To investigate the incidence of all skin cancers following primary MCC, a thorough search was conducted using the MEDLINE, Cochrane Central Register of Controlled Trials (CENTRAL), Australian New Zealand Clinical Trials Registry (ANZCTR), Web of Science Core Collection and EMBASE databases. The search was limited to studies with clearly defined population cohorts and employed keywords such as “Merkel cell carcinoma,” “non‐melanoma skin cancer,” “melanoma,” “second malignancies,” “second skin cancer,” “second primary skin cancer” and “primary Merkel cell carcinoma”. These terms were combined using Boolean operators “AND” and “OR”, as well as the search terms “risk ratio”. Additionally, the reference lists of relevant studies were examined in the literature. The search results were limited to studies involving humans (as shown in Figure 1).

### 2.2 Study Selection Criteria

The inclusion criteria for the studies examined were as follows: (i) studies published in English from January 1986 to July 2024; (ii) patients with all phases of MCCs; (iii) the incidence of second primary MCC cases was investigated; (iv) the analysis included studies that published risk ratios or provided data that enabled comparable conclusions to be drawn; (v) only original research articles were considered, with no consideration given to reviews, comments, letters or editorials; (vi) studies that reported the total number of patients with multiple primary tumours and second malignancies diagnosed within the cohort throughout the follow‐up period; (vii) studies with a confidence interval (CI) of at least 95% that reported either the expected cancer incidence rates in a matched background population or the rates of observed‐to‐expected malignancies; and (viii) studies in which melanomas and non‐melanoma skin cancer were specifically identified. The selection criteria excluded studies that mainly concentrated on reviews or pre‐clinical trials and did not have any results for second primary skin cancers.

### 2.3 Data Extraction and Statistical Analysis

As a part of our process, we thoroughly reviewed all relevant studies to identify those that met the inclusion criteria. We also resolved any discrepancies and cross‐referenced the data from multiple studies. In addition to extracting the paper's components (such as the first author's last name, country of study, study period, data source and other relevant information), we also recorded the number of primary MCC patients, their median age and the number of cases with a second skin cancer observed. The random‐effects model was employed for analysis, and risk ratios were used to represent the larger population of second skin cancer patients. These were reported with 95% CIs, and a *p* value of 0.05 or less was considered statistically significant when comparing risk ratios between groups. Corresponding forest plots were subsequently constructed, and we used the *I* ^2^ statistics to evaluate study heterogeneity. Depending on the *I* ^2^ values, we categorised the heterogeneity as low (0%–31%), moderate (31%–60%), substantial (61%–75%) or considerable (76%–100%). This categorisation reflects the percentage of variation across studies. To ensure the validity of our results, we employed Cochrane Review Manager Version 5.4.1 (Griffith University, Australia) to determine the combined risk ratios for the connection between all second skin cancers and other second cancers. We then conducted subgroup analyses based on various second skin cancers and geographical regions.

## 3 Results

**FIGURE 2:**
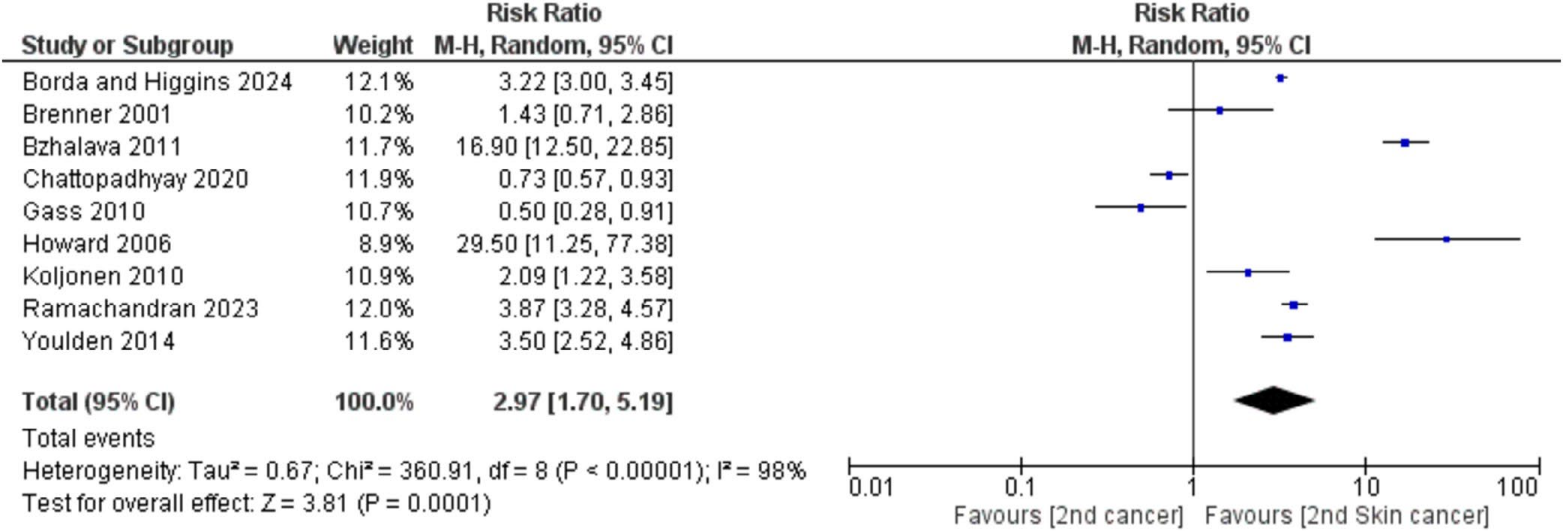
The potential risk of developing the second skin cancer compared to other second cancers following a primary diagnosis of Merkel Cell Carcinoma (MCC). The squares represent the risk ratios for each study, and the horizontal lines indicate the 95% confidence intervals. The size of each square reflects the relative weight assigned in the combined analysis, which employs a random effects model. The diamond symbol denotes the overall combined risk ratio, along with its corresponding 95% confidence interval. The vertical line indicates a risk ratio of 1, which signifies no effect.

The meta‐analysis included nine studies that met the inclusion criteria of a second skin cancer due to primary MCC [8, 12, 13, 14, 15, 16, 17, 18, 19]. These studies were conducted between 1958 and 2020 and were focused on primary MCC patients from various countries, including the USA, England, Finland, Denmark, Norway, Sweden, Israel and Australia. A total of 4626 cases of primary MCC were analysed across these studies. The risk ratio for developing the second skin cancer, as compared to other second cancers, varied from 0.50 (95% CI, 0.28–0.91) to 29.50 (95% CI, 11.25–77.38) (as shown in Figure 2). Most cases were reported in Europe among individuals of Caucasian ethnicity. The compiled data suggest that primary MCC is most commonly diagnosed in individuals over the age of 75, with the lowest incidence occurring in those aged 64 years (Table 1).

**TABLE 1:** A comprehensive review of the literature pertaining to documented cases of primary MCC resulting in a second skin cancer.

Country	Study period	Data source	Median age	Ethnic groups	All MCC cases	Primary MCC cases	Second skin cancer cases	Author
Israel	1983–1999	The Israel Cancer Register	73.0	Caucasian	67	17 (52.00% Male)	SCC: 7	Brenner et al.
USA	1986–2002	The Surveillance, Epidemiology, and End Results (SEER)	> 70.0	N/A	1306	122 (57.12% Female)	Melanoma: 4	Howard et al.
England	1995–2004	The Eastern Cancer Registration and Information Centre	79.0	N/A	67	27 (55.55% Male)	Melanoma: 2 SCC: 7 BCC: 9	Gass et al.
Finland	1979–2006	The Finnish Cancer Registry	> 75.0	Caucasian	172	34 (69.20% Female)	BCC: 11	Koljonen et al.
Denmark Norway Sweden	1980–2007 1990–2007 1990–2007	The Nordic Cancer Register	> 75.0	N/A	756	716 (60.00% Female)	Melanoma:6 (≥ 6 m) Melanoma:5 (≥ 1 year.) NMSC: 34 (≥ 6 m) NMSC: 5 (≥ 1 year.)	Bzhalava et al.
Australia	1982–2010	The Queensland Cancer Register	64.0	Most Caucasian	787	135 (65.00% Male)	Melanoma: 20 (≥ 2 m) Melanoma: 16 (≥ 1 year.) MCC: < 5 (≥ 2 m) MCC: < 5 (≥ 1 year.) Others: < 5 (≥ 2 m) Others: < 5 (≥ 1 year.)	Youlden et al.
Sweden	1958–2015	The Swedish Cancer Register	75.0	N/A	1011	140	Melanoma: 7 Invasive skin cancer: 38 In situ skin cancer: 7 SCC: 24 MCC: 5	Chattopadhyay et al.
USA	2000–2016	SEER	74.1	N/A	5578	575	Melanoma: 44 NMSC: 74	Ramachandran et al.
USA	2000–2020	The SEER‐22 registry	N/A	Caucasian (98.40%), Black (1.60%)	18 165	2860	Melanoma: 340 MCC: 302 Others: 36	Borda and Higgins

Abbreviations: BCC, basal cell carcinoma; MCC, merkel cell carcinoma; N/A, not applicable; NMSC, non‐melanoma skin cancer; SCC, squamous cell carcinoma.

### 3.1 A Comparison of the Second Skin Cancer and Other Second Cancers

A random effects model was chosen due to the considerable variation among the studies (*I* ^2^ = 98.00%). This model considers the possibility of unpublished or future studies that may not have been included in the meta‐analysis. The analysis revealed a risk ratio of 2.97 (95% CI, 1.70–5.19, *p =* 0.0001), indicating that the likelihood of developing a second skin cancer is significantly increased; specifically, the risk is 2.97 times greater compared to other second cancers (Figure 2). Even after removing extreme values of the risk ratio to lessen the impact of study‐specific biases, the data still show a significantly potential increase in the risk of developing the second skin cancer compared to other second cancers (data not shown).

### 3.2 Analysis of Different Types of the Second Skin Cancer

**FIGURE 3:**
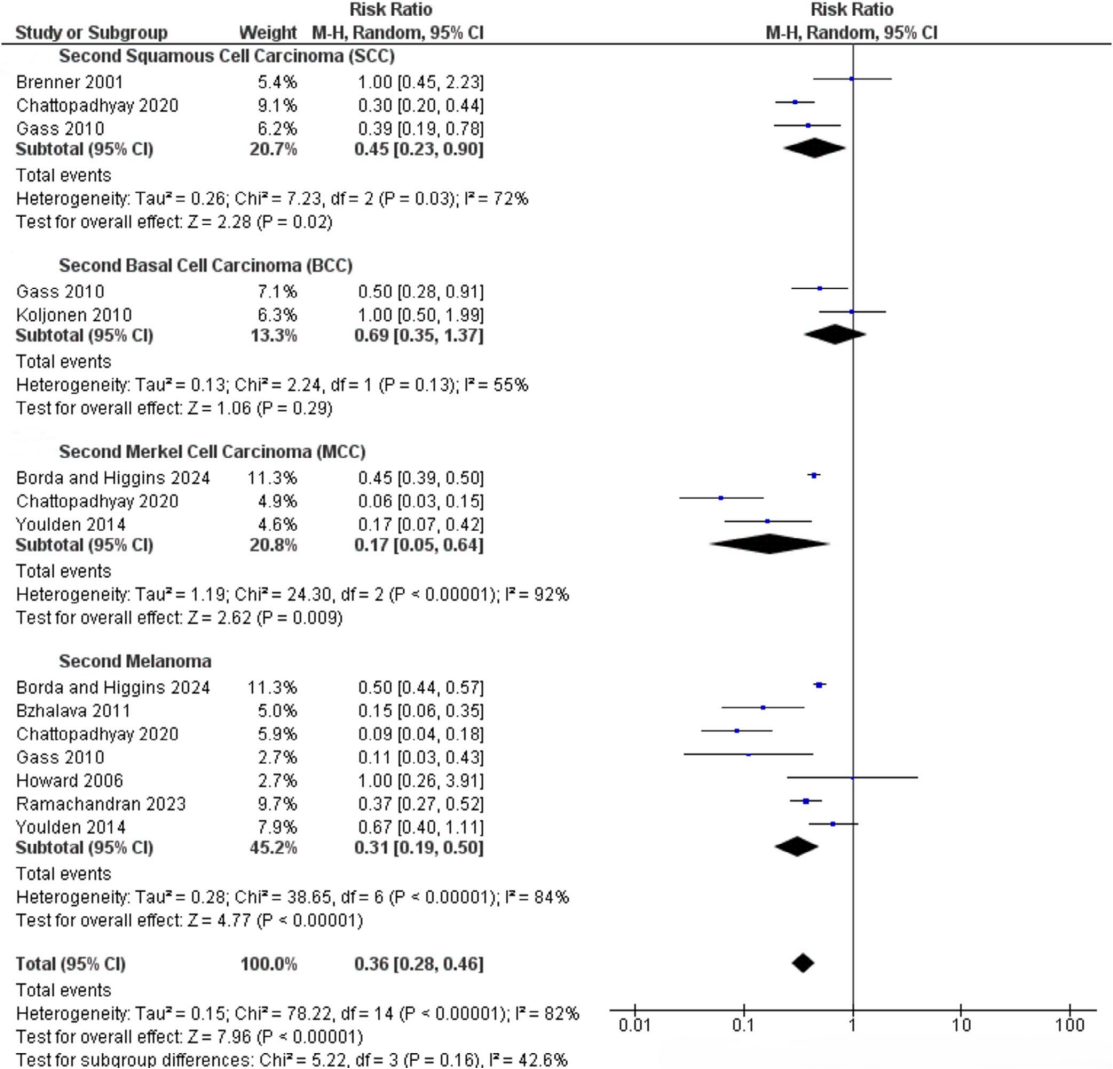
The risk of developing different types of second skin cancer following a primary Merkel cell carcinoma. Each square represents the risk ratio identified in individual studies, while the horizontal lines show the 95% confidence intervals associated with these ratios. The size of each square reflects the relative weight assigned to that particular study in the overall analysis, which utilises a random effects model. The diamond symbol indicates the overall combined risk ratio along with its corresponding 95% confidence interval. The vertical line marks a risk ratio of 1, which represents no effect.

The main analysis indicates that most patients with primary MCC are more likely to develop a second skin cancer compared to other second cancers. The population of the second skin cancer was then categorised into individual types, revealing a considerable amount of heterogeneity (*I* ^2^ = 84%). The random effects model was used to calculate the combined risk for all specific types of second skin cancer reported across all studies, which included SCC, BCC, melanoma and MCC itself. As shown in Figure 3, the overall risk ratio for each type of second skin cancer in patients with primary MCC compared to the general population was 0.36 (95% CI, 0.28–0.46). Among these cancer types, second BCC showed the highest risk ratio (0.69, 95% CI 0.35–1.37), followed by second SCC (0.45, 95% CI 0.23–0.90), second melanoma (0.31, 95% CI 0.19–0.50) and second MCC (0.17, 95% CI 0.05–0.64). However, the analysis revealed no significant difference for the second BCC.

Furthermore, it can be concluded that patients with primary MCC have a 1.9‐fold higher probability of developing second BCC, followed by second SCC (1.25‐fold) and second melanoma (approximately 1‐time risk). In contrast, the likelihood of developing the second MCC after having primary MCC is lower than that of the other skin cancers in the studies reported.

### 3.3 The Second Skin Cancer Within Geographic Areas

**FIGURE 4:**
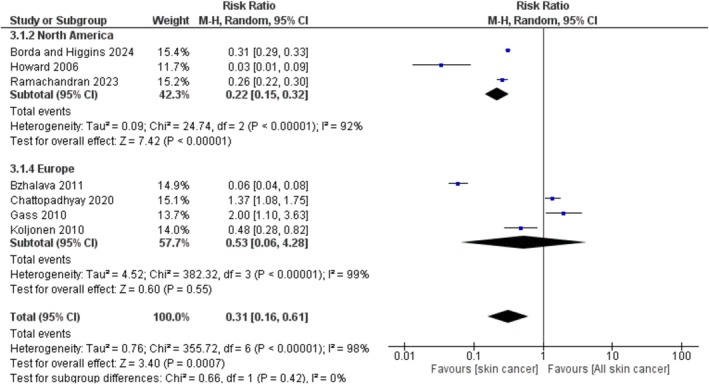
The risk of developing the second skin cancer following the initial development of MCC in different geographic areas. Each square denotes the risk ratio found in specific studies, while the horizontal lines represent the 95% confidence intervals related to these ratios. The size of each square indicates the significance of that particular study within the overall analysis, which uses a random effects model. The diamond symbol shows the overall combined risk ratio along with its related 95% confidence interval. The vertical line indicates a risk ratio of 1, signifying no effect.

We further conducted a subgroup analysis of the second skin cancer within specific geographical regions to assess the correlation between UV exposure and the incidence of the second skin cancer following primary MCC. The random effects model was employed to determine the combined risk for each country of study. Our analysis was limited to two primary continents: North America and Europe, given that studies from other continents were represented by only one study, which potentially introduces bias into the findings. The results from the subgroup analysis revealed that the overall risk ratio for certain regions, in comparison to general regions, is 0.31 (95% CI, 0.16–0.61). Among patients with the second skin cancer, those in Europe exhibited a higher risk ratio of 0.53 (95% CI, 0.06–4.28, *p* = 0.55) compared to their counterparts in North America (USA), who had a risk ratio of 0.22 (95% CI, 0.15–0.32, *p* < 0.00001). As shown in Figure 4, we concluded that Europe—particularly northern Europe, where the included studies were conducted—has a non‐significant 1.7 times greater likelihood of developing a second skin cancer compared to North America, which has a significantly lower likelihood of 0.7 times for developing the second skin cancer.

## 4 Discussion

This meta‐analysis aims to provide a comprehensive understanding of the relationship between primary MCC and the subsequent risk of developing additional skin cancers, as well as related demographic factors. Our analysis, which included 4626 cases of primary MCC, derived from nine studies conducted across various countries, has established that individuals with a history of primary MCC are at a significantly elevated risk for developing second skin cancer compared to those with other second cancers. Specifically, our findings reveal a 2.97‐fold increase in the likelihood of developing a second skin cancer, aligning with previous research that has observed comparable patterns [10], emphasising the necessity of close surveillance for other skin cancers in individuals with MCC.

Our analysis also indicates that the risk of developing different types of second skin cancer varies significantly. BCC was identified as the most frequently occurring second skin cancer, followed by SCC and melanoma. Notably, patients diagnosed with primary MCC exhibit a 1.9‐fold increased risk for BCC, suggesting a particular susceptibility to this form of cancer. This observation aligns with the findings of Koljonen et al. who reported that approximately one‐third of primary MCC were BCC. This could suggest a biological link that makes individuals more prone to BCC after being diagnosed with MCC. In this context, MCPyV, or Merkel cell polyomavirus, represents a recently identified human polyomavirus linked to the aetiology of MCC. In a relevant study involving 71 immunocompetent BCC patients, 39.4% indicated that 39.4% tested positive for MCPyV DNA, compared to 28.3% of 60 immunocompetent SCC patients; similarly, 47.1% of BCCs and 26.7% of SCCs among immunosuppressed patients tested positive for MCPyV DNA [20]. This could explain why our study observed a higher incidence of second BCC following second SCC and other second skin cancers for primary MCC.

Geographic disparities in the incidence of second skin cancer have also been observed, particularly between Europe and North America. The observed non‐significant 1.7‐fold increase in the risk of developing a second skin cancer in Europe may be attributed to higher levels of UV exposure, especially in northern regions where the population predominantly consists of individuals of Caucasian descent. Research indicates that individuals with red hair, light‐coloured eyes, north European ancestry and a predisposition to sunburn are at an increased risk for developing skin cancer, particularly BCC [21]. However, the association between UV exposure and MCC remains less thoroughly investigated compared to melanoma. A study on melanoma found that Northern Europe had a higher population‐attributable fraction (PAF) of melanoma cases related to UV radiation exposure when compared to North America (USA), with both regions having some of the highest UV levels in the world [22]. The link between UV exposure and skin cancer may be influenced by factors such as ozone depletion, urbanisation and variations in altitude and latitude [23]. Thus, the trends identified in our analysis may suggest a potential link between UV exposure and second skin cancer among patients with primary MCC. This association highlights the importance of implementing preventative sun protection measures and conducting regular dermatological evaluations for at‐risk populations.

While this meta‐analysis offers valuable insights, several limitations warrant attention. Firstly, considerable heterogeneity among the studies may affect the generalisability of our results due to variations in study design. Additionally, the potential for publication bias should be acknowledged; for example, our exclusion of non‐English studies may have constrained our findings. Since our analysis predominantly involved Caucasian populations, further research is essential to assess risks in more ethnically diverse groups. The geographic scope of this research was limited to four major continents—Europe, Asia (specifically Israel), North America and Australia/Oceania—potentially resulting in bias due to the omission of other regions. A notable constraint of our study is the extensive time frame it encompasses, spanning 62 years from 1958 to 2020. Nevertheless, notable advancements in cancer diagnostics and treatment, as well as heightened awareness regarding cancer prevention and the use of sunscreen, have emerged in recent years. Consequently, the prevalence of MCC and the second skin cancer may be substantial. Future studies should aim to explore the underlying mechanisms that contribute to the heightened risk of second skin cancer in MCC patients and consider a broader geographic representation to enhance the robustness of the findings. For instance, investigating the relationship between patients with Merkel cell polyomavirus (MCPyV), known to be associated with MCC, could yield valuable insights into the factors contributing to both primary MCC and the second skin cancer, particularly regarding whether MCPyV‐negative MCC is indeed associated with the second skin cancer. Despite these limitations, our meta‐analysis remains the most comprehensive and up‐to‐date investigation available, shedding light on the connection between primary MCC and second skin cancer. Standardised software was utilised for study retrieval, and meta‐analytical methods were applied for data synthesis.

In conclusion, our findings reveal a compelling association between primary MCC and an increased risk of developing second skin cancer, with BCC being the most prevalent among them. The need for proactive monitoring and preventative measures in this high‐risk group cannot be overstated. Further research is essential to unravel the complexities of this association and to refine clinical management strategies for patients with primary MCC.

## Author Contributions

Conceptualization: T.C., N.A.J.M.; Data Curation: T.C., N.A.J.M.; Formal Analysis: T.C., N.A.J.M.; Funding Acquisition: N.A.J.M.; Investigation: T.C.; Methodology: T.C.; Supervision: N.A.J.M.; Validation: T.C.; Writing – Original Draft Preparation: T.C.; Writing – Review and Editing: T.C., N.A.J.M.

## Conflicts of Interest

The authors declare no conflicts of interest.

## Data Availability

Data sharing not applicable to this article as no datasets were generated or analysed during the current study.
